# 3D STED Super-Resolution Imaging Strategy for Visualizing Synaptic Nano-architecture in Brain Cryosections

**DOI:** 10.21769/BioProtoc.5644

**Published:** 2026-04-05

**Authors:** James Scripter, Adam Skeens, Grace Jones, Yeasmin Akter, Martin Hruska

**Affiliations:** Department of Neuroscience, Rockefeller Neuroscience Institute, West Virginia University, Morgantown, WV, USA

**Keywords:** Tau-STED microscopy, Brain cryosections, PSD-95, Excitatory synapse, Nanobody, Paraformaldehyde, Glyoxal

## Abstract

Super-resolution imaging of synapses in intact brain tissue remains challenging because light scattering, photobleaching, and limited probe penetration, along with antigen accessibility within the densely packed postsynaptic densities (PSDs), constrain resolution and labeling efficiency. Here, we present a protocol utilizing thin brain cryosections and tau-stimulated emission depletion (STED) nanoscopy to visualize the intricate nano-architecture of excitatory synapses in situ. Slicing the brain into 6 μm sections allows for highly efficient and even penetration of probes throughout sections while ensuring that the resolution is not significantly impacted by the imaging depth of the tissue. We outline step-by-step instructions for labeling pre- and postsynaptic nano-architecture using antibodies and nanobodies, highlighting how fixative choice influences the labeling efficiency of synaptic proteins. While this protocol is compatible with both confocal and super-resolution imaging, when combined with rapid image acquisition times of tau-STED, it enables clear separation of key synaptic features in three dimensions with minimal photobleaching. Thus, this approach enables robust multiplex imaging of fluorescently labeled synaptic proteins in the brain, providing exceptional spatial resolution for visualization and quantification of synaptic nanoarchitecture in its native environment.

Key features

• Detailed protocol for in situ 3D STED microscopy with ~50 nm XY and ~100 nm Z resolution.

• Optimized strategies for labeling pre- and postsynaptic nano-architecture using antibodies and nanobodies, including guidance on fixative choice.

• Unified workflow for visualizing synaptic morphology and nanoarchitecture to uncover molecular synaptic diversity in the brain at the nanoscale.

## Graphical overview


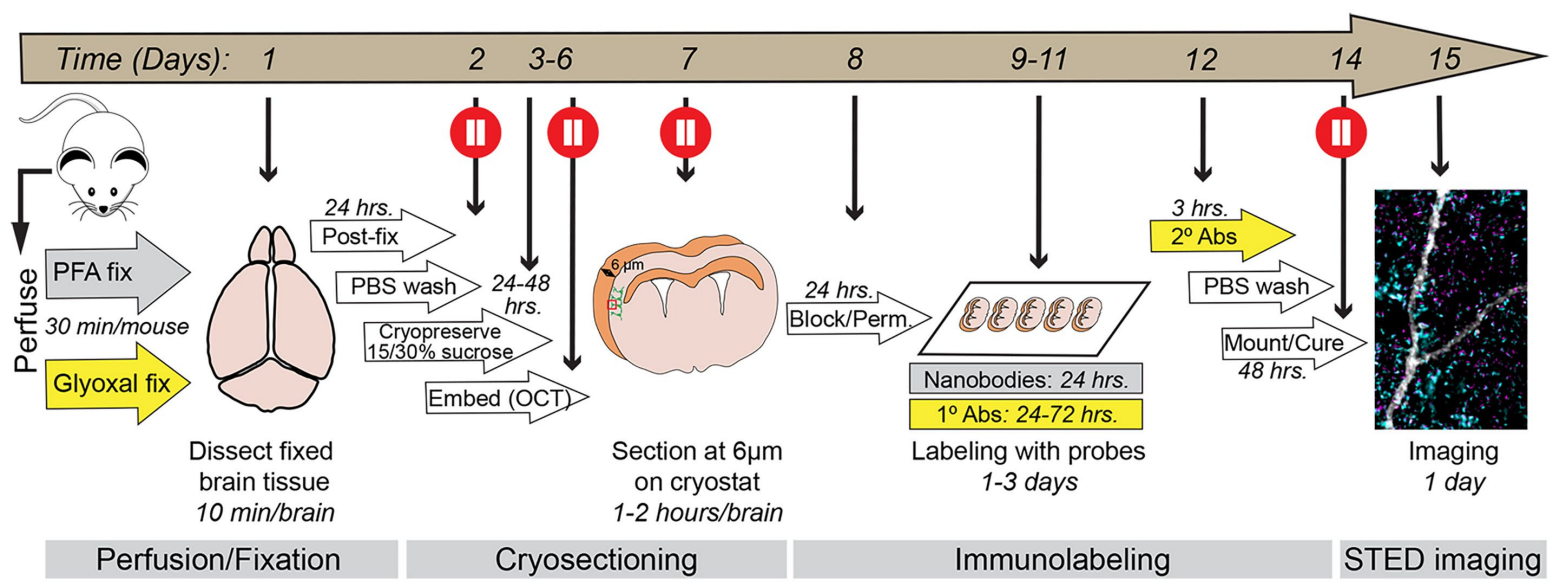


**Graphical overview of the procedure.** Schematic outlining each step of the protocol from day 1 to day 15. The timeline for each step is indicated. The protocol is divided into four parts: (1) Perfusion/fixation, (2) cryosectioning, (3) immunolabeling, and (4) imaging, each described in detail in the text below. The entire workflow takes approximately two weeks to complete and includes multiple pause points (red circles) where the procedure can be temporarily paused or where brain tissue can be safely stored.

## Background

Over the last decade, the development of diverse super-resolution imaging platforms has enabled the investigation of cell-biological questions with unprecedented detail. For the field of neuroscience and synaptic biology, in particular, super-resolution microscopy transformed our understanding of the molecular mechanisms that underlie synaptic transmission and plasticity—the fundamental processes that are key for learning and memory [1,2]. Yet, despite these advances, the methodology has been limited mainly to studies in primary neurons from the cortex and hippocampus [3–11]. However, the brain contains billions of neurons and trillions of synapses, organized into distinct anatomical layers and domains that encompass a vast diversity of synaptic subtypes with unique molecular, structural, and functional characteristics [12–16]. Therefore, achieving transformative discoveries in the molecular biology of synapses, in both health and disease, will require super-resolution imaging approaches that capture the nanoscale organization of synaptic proteins directly within their native brain environment [17,18].

Immunolabeling offers a robust way to examine the molecular complexity of synapses in the brain [19–21]. Yet, several challenges hinder the application of super-resolution microscopy in situ. First, the intricacies of brain tissue, with its high lipid content and dense neuropil, make it difficult to resolve molecules in sub-diffraction structures such as synapses with high precision [22,23]. Critically, resolution decreases with tissue depth [24]. Thus, combined with the limited penetration of large probes to the depths of brain tissue, this crucially limits super-resolution imaging in brain sections. Second, the postsynaptic densities (PSDs) of excitatory synapses are densely packed with thousands of distinct proteins, making molecules localized to this sub-structure notoriously hard to label with conventional reagents [25,26]. Third, paraformaldehyde fixation is known to mask antigens due to the high degree of cross-linking, further reducing probe affinity [27]. Altogether, these limitations reduce labeling efficiency, compromise the signal-to-noise (S/N) ratio, and leave weaker signals more susceptible to photobleaching.

In this protocol, we describe strategies to overcome these limitations by providing detailed instructions on how to achieve highly efficient immunolabeling of synapses in brain cryosections, thereby maximizing the ability to robustly identify synaptic nano-architecture in situ in three dimensions. The full workflow takes approximately two weeks and contains several steps, such as brain isolation, preparation of thin cryosections, and immunolabeling. Our primary focus is to provide guidance on tissue preparation, including the choice of fixative, and on selecting probes for efficient labeling of proteins in pre- and postsynaptic terminals. To this end, we highlight the use of small, single-domain nanobodies as an alternative to larger conventional antibodies, enabling highly multiplexed, high-resolution imaging of molecular nano-organization of synapses in brain tissue. We then describe the experimental steps and imaging configuration required to visualize synaptic nano-architecture in labeled brain sections using three-dimensional stimulated emission depletion (3D-STED) microscopy, with particular emphasis on the fluorescence lifetime imaging (FLIM)-based Leica Stellaris STED platform. Importantly, this protocol can be readily adapted to other super-resolution imaging approaches, including expansion microscopy [28]. Together, this protocol provides a unified framework for immunolabeling-based imaging of synaptic nano-architecture in situ.

## Materials and reagents

**Biological materials**

1. Mouse: B6. Cg-Tg (Thy1-YFP)16Jrs/J (Jackson Laboratory, Strain: 003782/ RRID: IMSR_JAX:003782) [29]

**Reagents**

1. 2-Methylbutane (Fisher Scientific, catalog number: O3551-4)

2. Acetic acid (Fisher Scientific, catalog number: A38S-500)

3. Antibodies and nanobodies (various suppliers, see Table S1)

4. Calcium chloride dihydrate (CaCl_2_·2H_2_O) (Fisher Scientific, catalog number: BP510-500)

5. Chromium (III) potassium sulfate (Sigma-Aldrich, catalog number: 60152-100G)

6. D-(+)-Glucose (Sigma-Aldrich, catalog number: G7021-1KG)

7. D-Sucrose (Fisher Scientific, catalog number: BP220-1)

8. EM-grade paraformaldehyde (PFA) (Polysciences, catalog number: 00380-1)

9. Ethanol 95% (v/v) (Decon Labs, Inc., catalog number: 2801)

10. Gelatin from bovine skin (Sigma Aldrich, catalog number: G9391-100G)

11. Glyoxal 40% (w/v) solution in water (Sigma-Aldrich, catalog number: 128465)

12. Heparin (Akron Biotech, catalog number: AK3004-5000)

13. Isoflurane (Piramal Pharma Limited, catalog number: NDC66794-017-25)

14. Ketamine (Ketaset Injectable C IIIN, 100 mg/mL) (Patterson Veterinary, catalog number: 07-803-6637; Schedule III DEA license required)

15. Normal goat serum (Gibco, catalog number: 16210-064)

16. Magnesium sulfate heptahydrate (MgSO_4_·7H_2_O) (Fisher Scientific, catalog number: BP213-1)

17. PBS, pH 7.4 (Thermo Fisher, catalog number: 10010023)

18. Potassium chloride (KCl) (Fisher Scientific, catalog number: BP366-500)

19. ProLong^TM^ glass antifade mountant (Thermo Fisher, catalog number: P36984)

20. Sodium bicarbonate (NaHCO_3_) (Sigma-Aldrich, catalog number: S5761-500G)

21. Sodium chloride (NaCl) (Fisher Scientific, catalog number: S271-500)

22. Sodium phosphate dibasic anhydrous (Na_2_HPO_4_) (Fisher Scientific, catalog number: BP332-500)

23. Sodium phosphate monobasic anhydrous (NaH_2_PO_4_) (Fisher Scientific, catalog number: BP329-1)

24. Tissue-Tek optimal cutting temperature (O.C.T) compound (Sakura FineTek USA, catalog number: 4583)

25. Triton X-100 (Sigma-Aldrich, catalog number: T9284-100ML)

26. Xylazine (AnaSed) injection 100 mg/mL (Patterson Veterinary, catalog number: 07-895-0792)

**Solutions**

1. Oxygenated artificial cerebrospinal fluid (ACSF), pH 7.4 (see Recipes)

2. 0.4 M phosphate buffer (PB) (see Recipes)

3. 4% (w/v) PFA solution, pH 7.4 (see Recipes)

4. 9% (v/v) glyoxal, 8% (v/v) acetic acid, pH 4 (see Recipes)

5. Gelatin solution (see Recipes)

6. Chromium potassium sulfate solution (see Recipes)

7. Blocking and permeabilization buffer (see Recipes)

**Recipes**

**1. Oxygenated ACSF solution, pH 7.4**

**Table:** 

Reagent	Final concentration	Quantity or volume
NaCl	125 mM	1.826 g
KCl	2.5 mM	0.046 g
CaCl_2_ (dihydrate)	2 mM	0.073 g
NaHCO_3_	25 mM	0.525 g
NaH_2_PO_4_	1.25 mM	0.037g
MgSO_4 _(heptahydrate)	2 mM	0.123 g
D-Glucose	10 mM	0.450 g
Heparin (191.5 U/mg)	10 U/mL	13 mg
Milli-Q H_2_O		Up to 250 mL
Total		250 mL

Oxygenate the solution on ice for 30 min to 1 h immediately prior to use. Afterward, adjust to pH 7.4, if needed. Keep ice-cold. Add heparin right before use. Prepare the solution fresh each time.

**2. 0.4 M PB**

**Table:** 

Reagent	Final concentration	Quantity or volume
Na_2_HPO_4_	320 mM	45.68 g
NaH_2_PO_4_	87 mM	10.49 g
Milli-Q H_2_O		Up to 1 L (800 mL before pH adjustment, rest after pH adjustment)
Total		1 L

The solution should be at pH 7.4 when prepared, and no pH adjustment should be necessary. This stock solution is used to prepare 0.1 M PB by diluting it 1:4 in Milli-Q water for other solutions or washing steps in the protocol. The stock can be stored at room temperature (RT) for several months.

**3. 4% (w/v) PFA solution, pH 7.4**

**Table:** 

Reagent	Final concentration	Quantity or volume
EM-grade PFA	4% (w/v)	20 g
0.4 M PB (Recipe 2)	0.1 M	125 mL
Milli-Q H_2_O		Up to 500 mL (300 mL before pH adjustment, rest after pH adjustment)
Total		500 mL

Heat approximately 300 mL of Milli-Q H_2_O to ~60 °C in a 500 mL beaker on a heated stir plate in a fume hood (do not exceed 65 °C). Once the water reaches temperature, add the PFA and allow it to dissolve while stirring for approximately 30 min; the solution will remain slightly cloudy at this stage. Slowly add 10 N NaOH dropwise (~10 drops) while stirring until the solution becomes completely clear. Add 125 mL of 0.4 M PB (Recipe 2) to the PFA solution and adjust the pH to 7.4 using a pH meter. Transfer the solution to a 500 mL 0.45 μm Nalgene rapid-flow filter and filter under vacuum. Bring the solution to a final volume of 500 mL with Milli-Q H_2_O and chill on ice. This solution should be prepared fresh before use and should be used ice cold.

**4. 9% (v/v) glyoxal, 8% (v/v) acetic acid, pH 4.0 (adapted from [27])**

**Table:** 

Reagent	Final concentration	Quantity or volume
Glyoxal 40% (w/v) solution in water	9% (v/v)	112.5 mL
Acetic acid	8% (v/v)	40 mL
0.1 M PB		Up to 500 mL (250 mL before pH adjustment, rest after pH adjustment)
Total		500 mL

This solution is very acidic due to the presence of acetic acid. A large amount of strong base (10 N NaOH) is required to bring the pH to 4. Chill on ice before using for perfusion. Prepare fresh each time.

**5. Gelatin solution**

**Table:** 

Reagent	Final concentration	Quantity or volume
Gelatin from bovine skin	5 mg/mL	4.5 g
Milli-Q H_2_O		Up to 900 mL
Total		900 mL

Use a heated stir plate to fully dissolve gelatin into the solution. Prepare fresh right before use.

**6. Chromium potassium sulfate solution**

**Table:** 

Reagent	Final concentration	Quantity or volume
Chromium potassium sulfate	1 mM	450 mg
Milli-Q H_2_O		Up to 30 mL
Total		30 mL

This solution should be prepared fresh right before use.

**7. Blocking and permeabilization buffer**

**Table:** 

Reagent	Final concentration	Quantity or volume
1× PBS	1×	9 mL
Normal goat serum	5% (v/v)	0.5 mL
10% (v/v) Triton X	0.5% (v/v)	0.5 mL
Total		10 mL

The solution can be stored at 4 °C for ~1 month.

**Laboratory supplies**

**Figure 1.**
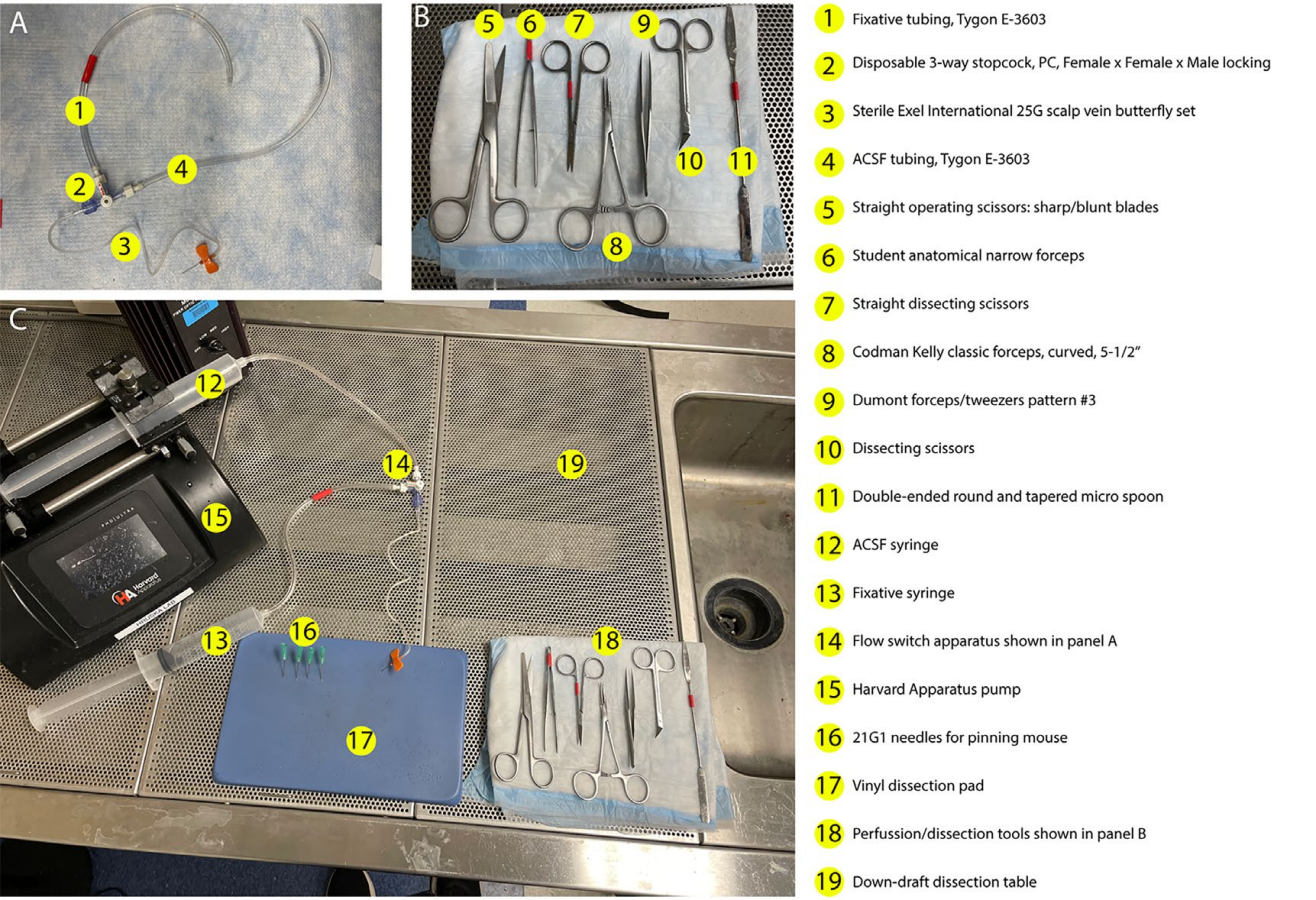
Setup for transcardial perfusion. (A) Assembly of the switching apparatus used to alternate flow between ACSF and fixative solutions, including tubing (#1 and #4), Lauer lock switch (#2), and a butterfly set (#3). (B) Layout of all perfusion and dissection tools required for the procedure (#5–11, see Laboratory Supplies no. 11 for additional detail). (C) Complete setup of perfusion workspace arranged for efficient workflow. Left: Pump with ACSF and fixative syringes connected to the switching apparatus (#12–13, #15). Center: Vinyl dissecting pad positioned centrally for stabilization of the animal (#17). Right: Full set of dissection instruments (#18) shown in detail in panel B.

1. Thermometer (-80 °C) (Fisher Scientific, catalog number: 13201640)

2. #1.5 rectangular coverslips (0.16–0.19 mm thick) (Fisher Scientific, catalog number: 22266882), gelatin-subbed (see section D for subbing procedure)

3. 10 cm Petri dishes (Fisher Scientific, catalog number: FB0875712)

4. 21 G × 1 in. Precision glide needle (BD, catalog number: 305165)

5. 2 L glass beaker (Fisher Scientific, catalog number: FB101-2000)

6. 60 mL disposable syringe non-sterile catheter tip (BD, catalog number: 301037)

7. Aluminum foil roll (FisherBrand, catalog number: 01-213-103)

8. Cotton ball McKesson medium non-sterile (Mckesson Medical-Surgical, catalog number: 980221)

9. CoverWell incubation chambers 22 mm × 40 mm × 0.2 mm (GraceBio, catalog number: 645402)

10. Disposable 3-way stopcock, PC, female × female × male locking (Cole-Parmer, catalog number: UX-48523-36)

11. Dissection tools (see Figure 1 for details)

a. Straight operating scissors: sharp/blunt blades (Figure 1, #5) (Fine Science Tools, catalog number: 14001-12)

b. Student anatomical narrow forceps (Figure 1, #6) (Fine Science Tools, catalog number: 91102-12)

c. Straight dissecting scissors (Figure 1, #7) (World Precision Instruments, catalog number: 14393)

d. Codman Kelly classic forceps, curved, 5-1/2′′ (Figure 1, #8) (Codman, catalog number: 32-4021)

e. Dumont forceps/tweezers pattern #3 (Figure 1, #9) (Stoelting, catalog number: 52100-03)

f. Dissecting scissors (curved) (Figure 1, #10) (Fine Science Tools, catalog number: 14082-09)

g. Double-ended round and tapered micro spoon (Figure 1, #11) (MicroSpatulas.com, catalog number: FD-21-401-10)

12. Falcon^TM^ 15 mL conical centrifuge tubes (Fisher Scientific, catalog number: 14-959-53A)

13. GATTA bead nanorulers (GATTAquant, Various distances, https://www.gattaquant.com/)

14. Kimwipes (KimTech, catalog number: 34120)

15. Low-profile microtome blades DB80 LS (Leica, catalog number: 14035843488)

16. Microscope slides: Fisherbrand^TM^ Superfrost^TM^ Plus stain slides (Fisher Scientific, catalog number: 22-034979)

17. Nalgene rapid-flow filters 0.45 μm (Thermo Scientific, catalog number: 166-0045)

18. Peel-A-Way disposable embedding molds 12 mm × 12 mm × 20 mm (Fisher Scientific, catalog number: 12-20)

19. Plastic staining rack (Mopec, catalog number: SP234)

20. Polyurethane ice bucket (Fisher Scientific, catalog number: 02-591-45)

21. Razor blades (Stanley, catalog number: 11-515)

22. Simport Scientific StainTray slide staining system (Simport Scientific, catalog number: 22-045-035)

23. Specimen disc (Leica, catalog number: 14047740044)

24. Sterile Exel International 25 G scalp vein butterfly set (Fisher Scientific, catalog number: 14-840-37)

25. Superfrost Plus microscope slides (Fisher Scientific, catalog number: 12-550-15)

26. Tygon E-3603 (Tygon, catalog number: AC00005)

27. United Scientific 100 mL stainless steel beaker (Fisher Scientific, catalog number: S139215)

28. Vinyl dissecting pad (Carolina, catalog number: 629006)

29. Wheaton Coplin staining jar (DWK Life Sciences, catalog number: UX-48585-20)

30. Zerostat anti-static instrument (Millipore Sigma, catalog number: Z108812)

## Equipment

1. Accumet AE150 Benchtop pH Meter (Fisher Scientific, catalog number: 13-636-AE150)

2. Down-draft table for perfusions (fume hood will suffice)

3. Standard Infuse/Withdraw PHD Ultra syringe Pump (Harvard Apparatus, catalog number: 70-3007)

4. Vetflo Traditional Anesthesia System with Vaporizer (Kent Scientific, model: 13-005-201)

5. Ultrasonic Liquid Processors (Misonix, model: XL-2000)

6. Leica CM3050 cryostat (Leica, model: CM3050S, reference number:14047033518)

7. Leica Stellaris 8 Confocal and tau-STED microscope system (Leica Microsystems, Mannheim, Germany)

## Software and datasets

1. LAS X (Leica, Version 4.8.2.295667, license required, available on Leica Stellaris 8 instruments)

2. Fiji (NIH, open-source platform for biological image analysis, ImageJ, Version 2.16/1.54P, free to download: https://imagej.net/software/fiji/downloads) [30]

3. Imaris (Oxford Instruments, Version 11.0.0, license required; free version from Imaris Viewer is available at: https://imaris.oxinst.com/imaris-viewer)

## Procedure

**A. Setup of the perfusion apparatus (Figure 1)**

1. Cut two 30 cm pieces of Tygon 3/32 in tubing (Tygon E-3603, #1 and #4 in Figure 1A).

2. Screw two connectors to the two female luer lock ends (#2 in Figure 1A), then attach the two 30 cm pieces of Tygon tubing from step 1 (Figure 1A).

3. Attach the Sterile Exel International 25G scalp vein butterfly set (#3 in Figure 1A) to the male luer lock end (#2 in Figure 1A).

4. Fill two 60 mL syringes for perfusion: one with the heparin-containing, ice-cold, oxygenated ASCF solution (#12 in Figure 1C) and the other with the fixative of choice (4% PFA or glyoxal, #13 in Figure 1C).

5. Attach the fixative syringe to the tubing designated for fixative (#1 in Figure 1A and #13 in Figure 1C).

6. Push the plunger to allow fixative to flow through the tubing and butterfly set.

7. Repeat steps A5–6 for the ACSF syringe (#4 in Figure 1A and #12 in Figure 1C), flushing the fixative out of the tubing and butterfly set with ACSF.

8. Place the ACSF syringe (#12 in Figure 1C) on the pump (#15 in Figure 1C).

9. Set up the dissection tools as shown below in Figure 1B.

10. Place the vinyl dissecting pad in the center of the workspace (#17 in Figure 1C).

11. Prepare anesthesia supplies in the fume hood.

**B. Transcardial perfusion**

*Note: For this protocol, we used Thy1-YFP-H mice from Jackson Laboratory. However, this procedure is applicable to any mouse strain of choice [29]. Below are key steps for achieving high-quality perfusion required for imaging with a high signal-to-noise ratio. For a more detailed description of the perfusion procedure, see [31].*

1. Anesthetize the mouse using cotton balls infused with isoflurane in an enclosed container (a large beaker covered with aluminum foil) until the animal cannot right itself (approximately 30 s).

2. Maintain anesthesia with an intraperitoneal injection of an overdose of ketamine (100 mg/kg) and xylazine (10 mg/kg).

**Caution:** Ketamine is a Schedule III controlled substance. Its use requires an active DEA license as well as appropriate institutional training for handling and disposal. As an alternative, anesthesia can be maintained using 4% isoflurane mixed with oxygen delivered via a VetFlo Traditional anesthesia system with a vaporizer, which does not require controlled-substance authorization.

3. When the mouse is unresponsive to a foot pinch, secure the anesthetized mouse to the vinyl dissection pad by inserting 21 G × 1 in. needles through the skin at the axilla and inguinal regions to immobilize all four limbs.

4. Sterilize skin on the abdomen area with 70% (v/v) ethanol.

5. Expose the peritoneal cavity by cutting through the skin and muscles along the midline of the abdomen up to the xiphoid process and then along the lower ribs with straight operating scissors (Figure 1B, #5).

6. The diaphragm should now be visible. Cut along the perimeter of the diaphragm to access the chest cavity using the straight dissecting scissors (Figure 1B, #7).

**Critical:** Take care not to nick or cut the heart, which lies just beneath the diaphragm on the left side, with the apex pointing toward the midline.

7. Expose the heart by cutting through the ribcage on the right and left sides and along the top, removing the ribs for better access to the pericardial cavity.

8. Using a hemostat (Figure 1B, #8), pinch the left ventricle at the apex of the heart and insert the 25 G needle of the Exel International scalp vein butterfly set (Figure 1A, #3) already connected via tubing to the 60 mL syringe containing ice-cold ACSF (as described in section A). Secure the needle in place by locking the hemostat.

9. Using clean, straight dissecting scissors (Figure 1B, #7), cut through the right atrium.

10. Begin perfusion with oxygenated, ice-cold, heparin-containing ACSF (Recipe 1) at the rate of 7 mL/min. Perfuse for approximately 2 min (total of ~14 mL). During this time, the dark red blood will be flushed from the heart and replaced by clear ACSF, indicating effective perfusion. The liver will lighten in color, and the lungs may become white.

11. Switch the flow from ACSF to 4% (w/v) PFA solution (Recipe 3) or 9% (v/v) glyoxal, 8% (v/v) acetic acid (Recipe 4) to begin fixing the brain tissue. Perfuse ~60 mL of fixative at the same flow rate as in step B10. Contraction and stiffening of the muscles and tail indicate a good quality fixation. Ensure the entire 60 mL runs through for complete fixation.

**Caution:** PFA is hazardous, so be careful handling it. Wear appropriate personal protective equipment (PPE).

**Critical:** Avoid introducing bubbles when switching from ACSF to fixative, as bubbles can impede perfusion and reduce fixation quality (see section A for proper setup).

**Critical:** PFA is less suitable for the labeling of synaptic proteins with antibodies due to antigen masking associated with extensive cross-linking. Glyoxal fixation significantly improves synaptic antibody labeling [27,28]. If nanobodies are being used, PFA fixation is sufficient and may be preferred when electron microscopy (EM) or correlative light-EM is planned (see General notes for additional detail) [32].

12. After perfusion is complete, decapitate the mouse using the straight operating scissors (Figure 1B, #5).

13. Using a scalpel or sharp razor blade, make a sagittal incision along the midline of the scalp from the snout to the back of the head to expose the skull.

14. Using the dissecting scissors (Figure 1B, #10) and forceps (Figure 1B, #9), carefully remove the skull to expose the entire brain, from the olfactory bulbs to the cerebellum.

15. Gently remove the brain by sliding a micro-spoon/spatula (Figure 1B, #11) underneath it and severing the cranial nerves. Take care not to tear the tissue.

16. Place the brain in a 15 mL conical tube containing 10–14 mL of the same fixative [4% (w/v) PFA or glyoxal] used for perfusion. Incubate (post-fix) the dissected brain in the fixative solution overnight at 4 °C to ensure complete fixation.

17. The next day, remove the fixative and replace it with 10 mL of 1× PBS. Incubate for 5–10 min at RT with gentle agitation. Repeat twice more, for a total of three PBS washes.

**Caution:** Ensure the proper disposal of the fixative according to environmental, health, and safety protocols of the institution.

**Pause point:** After the final wash, brains can be stored short-term (up to one week) in 1× PBS at 4 °C.

**C. Embedding**

1. Cryoprotect the whole brain by immersing it in 15% (w/v) sucrose in 0.1 M phosphate buffer (PB) and incubating overnight at 4 °C. Complete infiltration of sucrose is indicated when the brain sinks to the bottom of the tube, reflecting replacement of tissue water with the denser sucrose solution, which reduces the formation of ice crystals. Once the brain has fully sunk, transfer it to 30% (w/v) sucrose in 0.1 M PB and incubate at 4 °C until it again sinks to the bottom of the tube, which typically requires several days. The sinking of the brain to the bottom indicates complete sucrose infiltration.

2. Prepare the embedding setup. Fill a medium-to-large ice bucket halfway with dry ice and place a 100 mL stainless steel beaker in the center, ensuring the dry ice completely surrounds it. Pour 50–60 mL of 2-methylbutane into the beaker and allow it to reach -70 to -80 °C. Verify the temperature using a thermometer.

3. Fill the 12 mm × 12 mm × 20 mm embedding molds halfway with OCT compound.

4. Remove the brain from the 15 mL tube by pouring the 30% (w/v) sucrose and brain into a 10 cm Petri dish. Using flat-tip forceps, gently lift the brain and gently place it on a Kimwipe to remove excess moisture.

5. Place the brain, olfactory bulbs facing down, into the embedding mold filled halfway with OCT.

6. With the brain in a vertical position, fill the remainder of the mold with OCT compound.

**Critical:** Avoid creating bubbles, as they will affect freezing quality and subsequent cryosectioning.

7. Adjust the brain’s vertical orientation using the micro-spoon/spatula.

8. Using long forceps, carefully place and hold the mold containing the brain in the 2-methylbutane at -70 °C.

**Critical:** Do not fully submerge the mold in 2-methylbutane and avoid any direct contact between the brain tissue and the 2-methylbutane.

9. Allow the OCT to fully solidify (transitioning from a transparent liquid to a white solid), which takes about 2–3 min. Wrap the embedded brain with the mold in aluminum foil and store at -80 °C until ready for cryosectioning.

**Pause point:** Embedded brains can be stored long-term at -80 °C (for years).

**D. Subbing (coating) #1.5 rectangular coverslips**

*Note: For super-resolution microscopy, sections should be mounted directly onto 24 mm × 60 mm, #1.5 coverslips (0.16–0.19 mm), rather than slides, to facilitate imaging with high numerical aperture (NA, 1.4), low working distance (0.13 mm) objectives, such as the 100× STED lens [28]. To prevent brain sections from detaching during subsequent staining and washing steps, coverslips are coated (subbed) with gelatin. Alternatively, coverslips may be coated with poly-L-lysine (PLL), as described in [33].*

1. Prepare gelatin (Recipe 5) and chromium potassium sulfate (Recipe 6) solutions.

**Caution:** Chromium potassium sulfate is toxic. Handle it with care using proper PPE, and dispose of it according to institutional environmental, health, and safety protocols.

2. Place #1.5 microscope coverslips into plastic staining racks, ensuring both sides are exposed to the subbing solutions.

3. Place the racks with coverslips into a 2 L glass beaker containing enough 95% (v/v) ethanol to fully submerge them. Sonicate submerged coverslips for 15 min.

4. Remove the coverslips from the 95% (v/v) ethanol, place them on paper towels, and allow to air-dry.

5. Mix the gelatin (Recipe 5) and chromium potassium sulfate (Recipe 6) solutions in a 1:1 ratio.

6. Carefully place the dried coverslips into the gelatin and chromium potassium sulfate mixture and gently swirl for 1 min.

7. Remove the coverslips from the solution and wick away excess liquid with a Kimwipe. Allow them to dry at RT overnight. After drying, coverslips can be stored in their original boxes at room temperature for years.

**E. Cryosectioning (coronal sections)**

**Figure 2.**
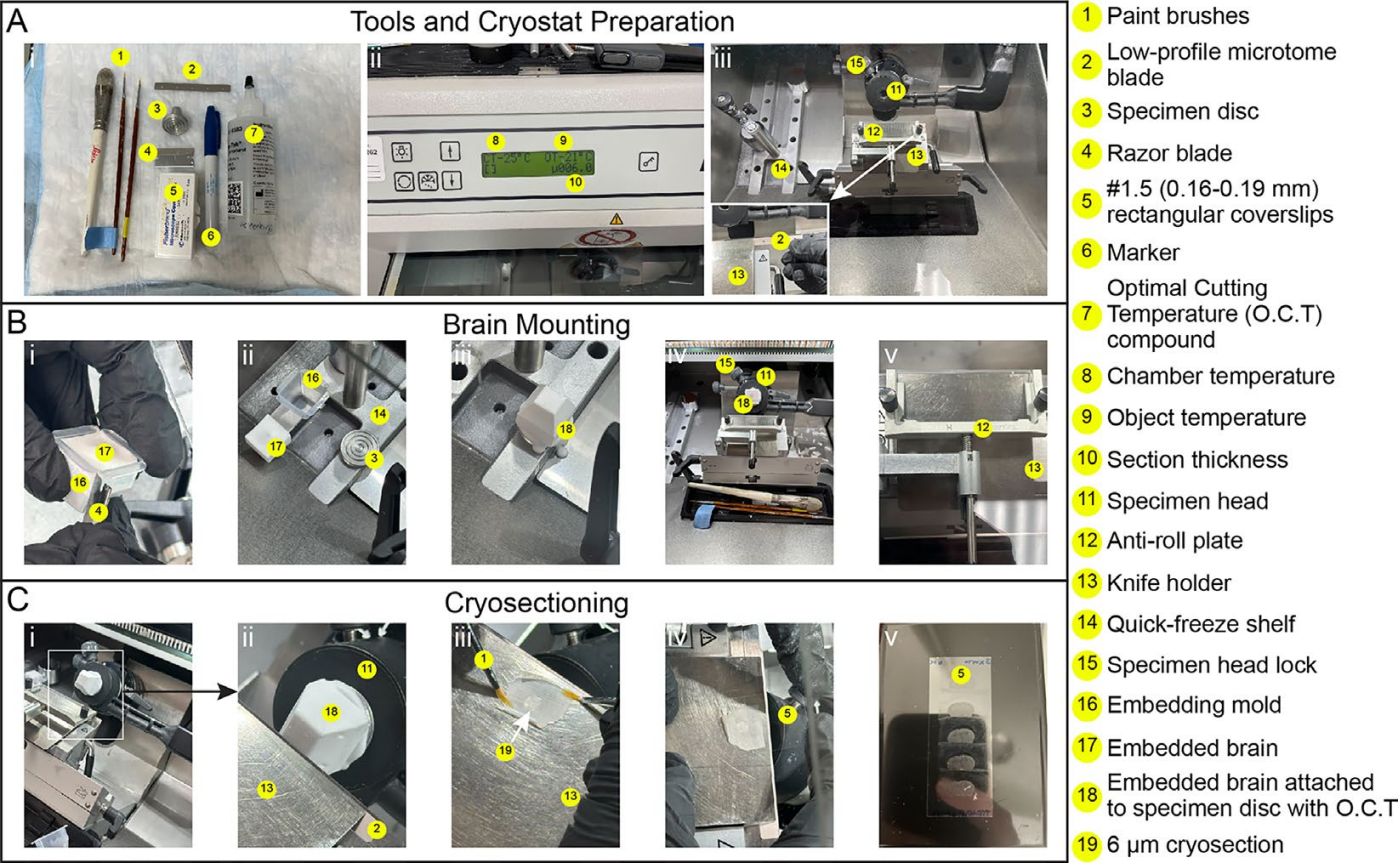
Cryosectioning workflow. (A) Tools and cryostat preparation: (i) Layout of tools required for cryosectioning (#1–7). (ii) Cryostat control display showing chamber temperature (-25 °C) (#8), object temperature (-21 °C) (#9), and section thickness (6 μm) (#10). (iii) Cryostat chamber layout (#11–15). (Inset) Placement of the low-profile microtome blade (#2) into the knife holder (#13). (B) Brain mounting: (i) Removal of the embedded brain (#17) from the embedding mold (#16), using a razor blade (#4). (ii) Placement of the specimen disc (#3) onto the quick-freeze shelf (#14). (**iii**) Attachment of the embedded brain to the specimen disc using O.C.T. compound (#18). (iv) Mounted specimen (embedded brain) secured in the specimen head (#11) and locked with the specimen head lock (#15). (v) Alignment of the anti-roll plate (#12) with the low-profile microtome blade (#2) in the knife holder (#13). (C) Cryosectioning: (i) Mounted specimen positioned away from the blade. (ii) Specimen advanced toward the blade (#2) secured in the knife holder (#13). (iii) A 6 μm cryosection (#19) being flattened using two paint brushes (#1). (iv) Collection of a 6 μm section onto a #1.5 rectangular coverslip (#5). (v) Coverslip (#5) containing collected brain sections.

1. Remove the embedded brain from -80 °C and place it at -20 °C 12 h prior to sectioning.

2. Set the cryostat object temperature (OT) to -21 °C and the chamber temperature (CT) to -25 °C (Figure 2A, ii).

3. Insert a low-profile blade into the cryostat knife holder and allow it to cool for at least 15 min (Figure 2A, iii).

**Caution:** Cryostat blades are extremely sharp. Keep your hands behind the anti-roll plate once the blade is secured in the knife holder.

4. Remove the embedded brain from the mold by cutting the mold open with a razor blade, then place the brain into the cryostat chamber (Figure 2B, i, ii).

5. Insert a specimen disc into one of the spaces on the quick-freeze shelf inside the chamber and cover the base of the disc with OCT compound to create a flat surface for mounting the brain (Figure 2B, ii).

6. Using forceps, place the brain (olfactory bulbs facing up) onto the specimen disc and allow the OCT to freeze, securing the brain in place (Figure 2B, iii).

7. Lock the specimen disc with the attached brain onto the specimen head of the cryostat (Figure 2B, iv).

8. Advance the specimen head until the tissue is just in front of the blade (Figure 2C, i, ii). Adjust the specimen head angle to center the brain relative to the blade for even sectioning. Using a razor blade, carefully trim away excess OCT surrounding the tissue to facilitate smooth cutting.

9. Set the cryostat trimming thickness to 30 μm and begin trimming, using the mouse brain atlas to guide progress to the desired brain area. For the somatosensory cortex (S1), trim along the anterior/posterior (A/P) axis until reaching approximately -1.05 mm from bregma.

10. During trimming, keep adjusting the specimen head angle to ensure even sectioning across the left and right hemispheres. Also, adjust the anti-roll plate to ensure sections are cut as flat sheets without curling, shredding, or bubbling.

11. Once the desired region is reached, set the cryostat to cut 6 μm sections and begin collecting cryosections onto gelatin-coated coverslips, as described in section D (Figure 2C, iii–v).

**Critical:** Ensure sections are free of tears, bubbles, and folds. Verify section quality under a transmitted-light microscope.

**Pause point:** Cryosections collected on glass coverslips can be stored dry at -80 °C for several months in slide boxes sealed with parafilm.

**F. Immunolabeling and mounting**

**Figure 3.**
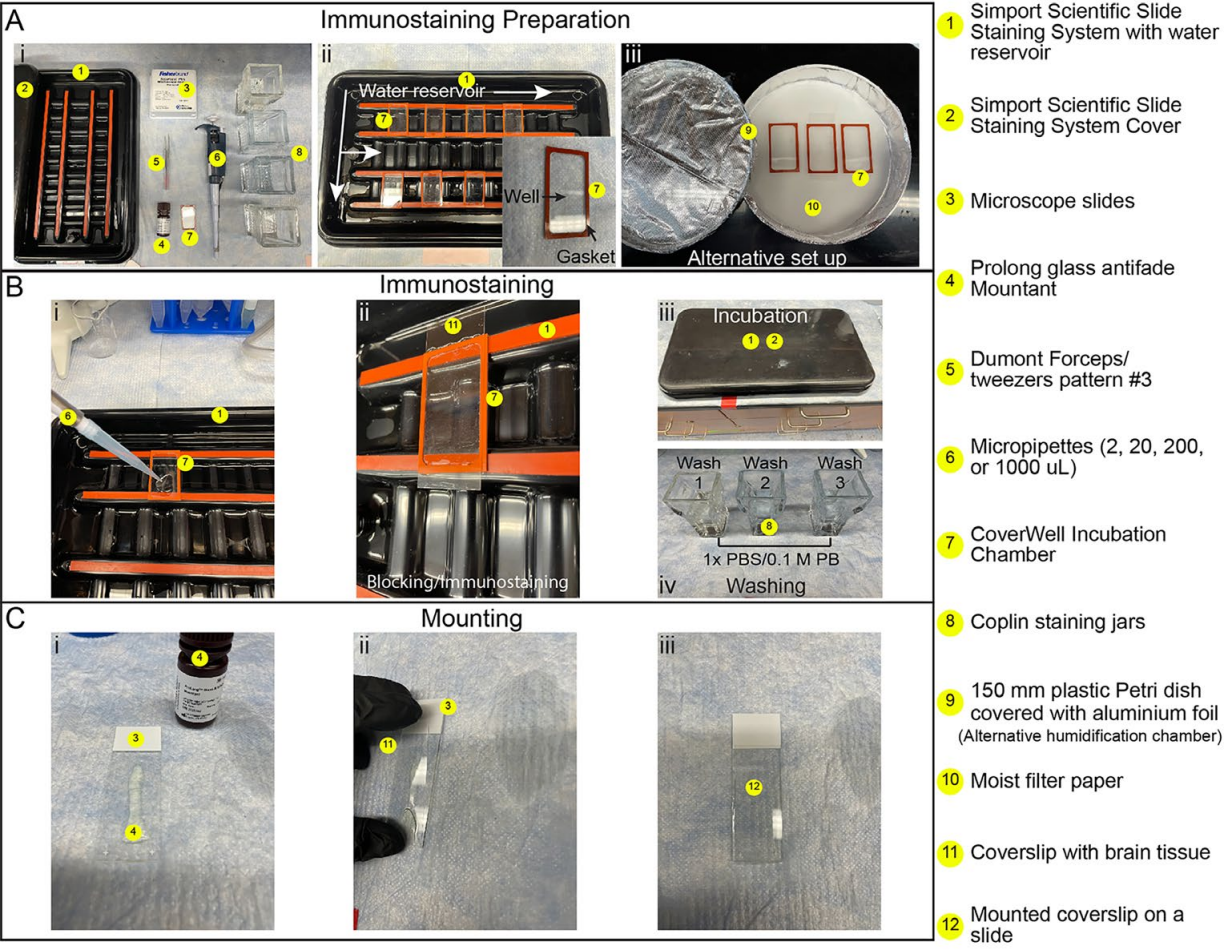
Immunostaining and mounting workflow. (A) Immunostaining preparation: (i) Layout of tools required for immunostaining and mounting (#1–8). (ii) Simport Scientific Slide Staining System filled with water (#1), containing multiple CoverWells positioned with gaskets facing upward (#7). (Inset) Close-up of a CoverWell (#7) showing the rubber gasket and central well for blocking and immunostaining solutions. (iii) Alternative staining setup consisting of an aluminum-covered 15 cm Petri dish (#9), with the moist filter paper (#10) on the bottom. (B) Immunostaining: (i) Addition of blocking or immunostaining solution to a CoverWell, with the gasket side facing up (#7), in the Simport Slide Staining System (#1). (ii) Coverslip with brain tissue (#11), placed onto the CoverWell (#7), containing the blocking or labeling solution. (iii) Simport Slide Staining System, covered to protect samples from light during incubations (#1, 2). (iv) Coplin jars (#8), filled with 1× PBS or 0.1 M PB for washing coverslips. (C) Mounting: (i) Application of ProLong Glass antifade mountant (#4) along the center of a microscope slide (#3). (ii) Coverslip with brain tissue (#11), lowered onto the slide at a ~45° angle to minimize bubble formation. (iii) Fully mounted coverslip with brain tissue free of air bubbles.

*Note: All blocking and immunolabeling steps are performed in a Simport Scientific Slide Staining System containing a bottom water reservoir to maintain humidity, thereby preventing evaporation, while providing a darkened environment to minimize photobleaching from ambient light (Figure 3). The use of CoverWell chambers (300 μL capacity) containing a gasket that forms a tight seal with the coverslip further reduces evaporation (Figure 3A, ii). As an alternative to the Simport Scientific Slide Staining System, a 150 mm Petri dish lined with moist filter paper can be used to hold coverslips/CoverWells during staining (Figure 3A, iii).*

1. Partially fill the bottom reservoir of a Simport Scientific Slide Staining System with distilled water and place it on a flat surface (Figure 3A, ii).

2. For each coverslip with mounted brain sections, place a CoverWell incubation chamber into the humidified Staining System, gasket side up (Figure 3B, i). As an alternative to using a CoverWell, a hydrophobic barrier can be drawn around the tissue using a PAP pen to allow blocking and staining in small volumes. However, the use of CoverWells is preferred, as they further reduce evaporation, especially during prolonged incubation periods.

**Critical:** Ensure that no water from the humidity chamber contacts the gasket of the CoverWell chamber. Moisture will prevent the gasket and coverslip from forming a tight seal, causing solutions to leak.

3. Pipette 300 μL of blocking buffer (Recipe 7) into the center of the CoverWell chamber (Figure 3B, i).

4. Carefully lay the coverslip, tissue side down, onto the liquid in the CoverWell chamber, ensuring that the buffer spreads evenly and no air bubbles form over the tissue (Figure 3B, ii).

5. Cover the humidified slide staining system and place it at 4 °C overnight (Figure 3B, iii).

6. Prepare primary antibodies or nanobodies in blocking buffer (Recipe 7) at the desired concentration (see Table S1 for validated synaptic antibodies and nanobodies).

7. Place clean CoverWell incubation chambers into a Slide Staining System and pipette 300 μL of the primary antibody/nanobody solution into the center of each chamber (Figure 3B, i).

8. Gently remove the coverslips from the blocking chambers. Remove excess blocking buffer by touching the coverslip edges to Kimwipes, then place the coverslips onto new CoverWell chambers containing the antibody/nanobody solution (Figure 3B, ii).

9. Incubate the coverslips at 4 °C in the Slide Staining System for 48 h (Figure 3B, iii).

**Pause point:** Coverslips can remain in primary antibody solution for up to 72 h at 4 °C.

10. Fill a Coplin jar with enough 1× PBS to fully submerge the coverslips and incubate for 5 min at RT. Repeat two more times for a total of three 5-min washes (Figure 3B, iv).

**Critical:** Avoid agitation. Excessive agitation can cause brain sections to detach from the coverslip.

11. After the final wash, incubate the coverslips for 2–3 h at RT in CoverWell chambers containing 300 μL of secondary antibodies (if needed) diluted to the desired concentration in blocking buffer (Recipe 7; see Table S1 for secondary antibody dilutions conjugated to compatible STED dyes).

12. Wash the coverslips in Coplin jars containing 1× PBS, performing three 5-min incubations at RT (Figure 3B, iv).

13. Following the final wash, incubate coverslips at RT for 5 min in 0.1 M PB (Figure 3B, iv; see preparation of Recipe 2 for details).

14. Remove the coverslips from the PB solution and air dry the tissue for 1–2 min.

**Critical:** A brief drying period helps prevent air bubble formation, which interferes with high-resolution imaging. Do not allow the brain sections to dry completely.

15. Add 200 μL of Prolong Glass antifade mountant directly onto a labeled microscope slide (Figure 3C, i).

16. Carefully place the coverslip with cryosections onto the slide, starting at the long edge and gently lowering it so that the mountant spreads evenly across the tissue (Figure 3C, ii, iii).

**Critical:** Avoid trapping air bubbles between the coverslip and slide. Air bubbles will dry out the tissue and render those areas unimageable.

17. Let the mounting medium cure in a dry dark area at RT for at least 48 h before imaging. For longer storage, seal the edges of the coverslip with nail polish after curing and store slides in a dark, dry, cold location (4 °C).

**G. Imaging and image processing**

**Figure 4.**
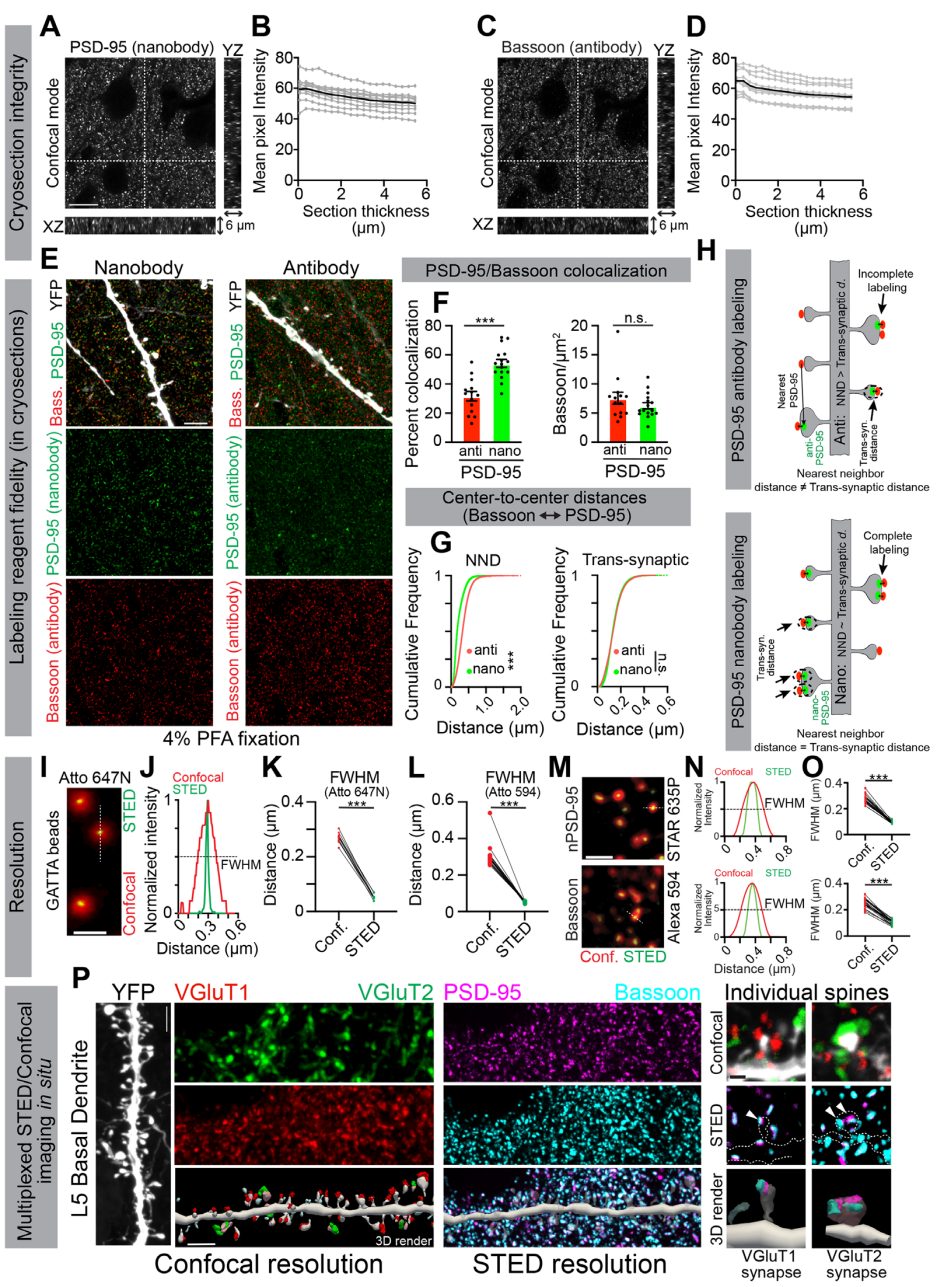
Validation of synaptic labeling strategies and 3D STED imaging in brain cryosections. (A–D) **Validation of cryosection integrity.** Representative confocal images of 6 μm cortical cryosections labeled with the PSD-95 nanobody (A) or Bassoon antibody (C). Orthogonal XZ and YZ views show cluster labeling intensity across the full thickness of the section. Quantification of mean cluster intensities at different depths in 10 independent cryosections (gray lines), with the overall average shown by the black line, demonstrating ~10% changes in the intensity from top to bottom of PSD-95 (B) and Bassoon (D) labeled cryosections. Scale bar for A and C, 10 μm. **Validation of labeling reagent fidelity:** (E) Representative two-channel tau-STED images of PSD-95 (green) labeled with either a nanobody or an antibody, together with Bassoon (red), along the apical dendrite of a layer 5 (L5) pyramidal neuron in 4% PFA-fixed cryosections from Thy1-YFP-H mice. YFP (gray), enhanced by anti-GFP labeling, was imaged in conventional confocal mode. Scale bar, 3 μm. PSD-95 nanobody labeling reveals numerous clusters, most of which colocalize with Bassoon. For PSD-95 antibody labeling, only a subset of PSD-95 clusters is clearly visualized. (F) Quantification of PSD-95/Bassoon colocalization shows significantly higher colocalization when PSD-95 is detected with a nanobody (n = 16 images, dots on the graph) compared to an antibody [n = 14 images (dots on the graph); ***p < 0.0001, unpaired two-tailed Student’s t-test]. Bassoon nanocluster density (clusters/μm^2^) does not differ between conditions (p = 0.2518). (G) Cumulative frequency distributions of all nanocluster pairs in the field demonstrate a tighter spatial relationship (nearest neighbor distance, NND) between nanobody-labeled PSD-95 and Bassoon nanoclusters (n = 39,405 cluster pairs) compared to the antibody-labeled PSD-95 and Bassoon (n = 68,806 cluster pairs; ***p < 0.0001, Kolmogorov–Smirnov test). Right: Cumulative frequency distributions of center-to-center distances of only colocalized cluster pairs (putative trans-synaptic partners). Despite fewer trans-synaptic pairs identified with PSD-95 antibody (n = 12,992 cluster pairs) compared to PSD-95 nanobody (n = 26,092 cluster pairs), there were no significant differences in centers of PSD-95 and Bassoon for either of the reagents (p = 0.4483, Kolmogorov–Smirnov test). Thus, the PSD-95 antibody can identify transynaptic nanocluster pairs but with much lower frequency. (H) Schematic illustrating nearest-neighbor distances (NND) and trans-synaptic distances between PSD-95 Bassoon nanoclusters in PFA-fixed sections. PSD-95 nanobody can identify transynaptic PSD-95/Bassoon pairs with high fidelity. While PSD-95 antibody can identify trans-synaptic pairs, often, the nearest Bassoon is too far away to be a trans-synaptic partner. Data about individual cluster distances were plotted as cumulative probability distributions and compared using a Kolmogorov–Smirnov test, as these nonparametric data do not follow a normal distribution. Bars represent the mean ± S.E.M. of normally distributed data. Scale bar in E, 3 μm. **Validation of STED resolution:** (I) Confocal (red) and STED (green) images of Atto 647N-labeled GATTA 50R beads were acquired with the sequence 2a configuration in Table 1. (J) Fluorescent width at half maxima (FWHM, dotted line) of confocal (red trace) and STED (green trace) modalities were determined along the white dotted line in I. (K, L) Confocal and STED FWHM for Atto 647N labeled GATTA beads (confocal: 270 nm ± 6 nm, STED: 54 nm ± 3 nm, ***p < 0.0001, paired two tail Student’s t-test, n = 10 beads) and for Atto 594 labeled GATTA beads (confocal: 319 nm ± 1.5 nm, STED 54 nm ± 1 nm, ***p < 0.0001, n = 18 beads). (M) Confocal (red) and STED (green) images of PSD-95 (top image, labeled with PSD-95 nanobody conjugated to Abberior STAR 635P, sequence 2 configuration in Table 1) and Bassoon (bottom image, labeled with the secondary antibody conjugated to Alexa Fluor 594, sequence 3 configuration in Table 1) in 6 μm brain cryosections. (N) FWHM of confocal (red trace) and STED (green trace) modalities determined along a single PSD-95 (top graph) and Bassoon (bottom graph) clusters (white dotted lines in M). (O) STED significantly improved resolution compared to confocal as determined by FWHM for both PSD-95 (top graph, confocal: 273 nm ± 33 nm, STED: 95 nm ± 9 nm, n = 22 clusters, p < 0.0001 paired, two-tailed Student’s t-test) and Bassoon (bottom graph, confocal: 258 nm ± 35 nm, STED: 113 nm ± 17 nm, n = 21 clusters, p < 0.0001 paired, two-tailed Student’s t-test). The graphs show paired comparisons of confocal and STED resolution for each bead or nanocluster, with each dot representing an individual bead or nanocluster measurement. Scale bars in I, 500 nm; in M, 1 μm. **Validation of multi-channel confocal/STED imaging in situ:** (P) Representative images of YFP-labeled dendritic spines from basal dendrites of L5 pyramidal neurons in 4% PFA fixed cryosections collected from the somatosensory cortex (S1) of Thy1-YFP-H mice. YFP (gray, enhanced by GFP antibody labeling), VGluT1 (red), and VGluT2 (green) were imaged by confocal microscopy; PSD-95 (magenta, labeled using nanobody) and Bassoon (cyan) were imaged with tau-STED. 3D reconstructions (Neurolucida 360) were used to classify VGluT1^+^ and VGluT2^+^ spines and map PSD-95 and Bassoon nano-architecture. Scale bar, 2 μm. Right: Insets showing individual spines with molecularly identified (VGluT1^+^ or VGluT2^+^) inputs and reconstructed nano-architecture. Scale bar, 1 μm. Data and images were adapted from [28].

*Note: The tissue preparation described in this protocol is compatible with both confocal and STED microscopy [28]. Depending on the instrument configuration and the number of available STED lines, 1–4 simultaneous STED channels can be achieved [6,28,34]. Here, we outline the steps for performing multi-channel, 3D STED imaging with simultaneous confocal microscopy to resolve synaptic nano-organization in dendritic spines receiving molecularly identified presynaptic inputs (Figure 4). All imaging is performed on a Leica Stellaris 8 tau-STED system, equipped with a tunable white-light laser (WLL) for excitation and continuous-wavelength (CW) 592 nm, CW 660 nm, and pulsed 775 nm STED lines.*

1. Prior to STED imaging, ensure proper alignment of the STED lasers relative to the WLL excitation using reflective 80 nm gold particles.

**Critical:** The center of the excitation laser must pass precisely through the hole of the STED donut. A deviation of more than 20–25 nm will negatively affect image quality and may cause the loss of super-resolution altogether.

2. Target proteins should be labeled (directly or indirectly) with STED-compatible fluorophores, and acquisition should follow the sequence order shown in Table 1 [35]. The pulsed 775 nm depletion laser is compatible with most fluorophores in red and near infra-red excitation spectra and enables the highest resolution in the XY plane (~50 nm) [6,28]. Therefore, whenever possible, proteins most critical to the experimental design should be imaged in this spectral range. The CW 660 nm and CW 592 nm STED beams achieve sub-diffraction resolution of ≥80 nm in the XY plane [34]. These lasers can be used simultaneously with the 775 nm STED laser, following the acquisition order from the most red-shifted dyes first to the most blue-shifted dyes last, as outlined in Table 1.

**Table 1.** Imaging configuration for simultaneous five-channel 3D STED and confocal acquisition

Order of image acquisition	Fluorophore	Excitation line (AOBS%)	Tau-STED depletion line (AOBS%)
Sequence 1	Alexa Fluor 790	750 nm (20%, confocal only)	N/A
Sequence 2	Abberior STAR 635P	635 nm (15%)	Pulsed 775 nm (10%)
Sequence 2 (alternate)	Atto 647N	640 nm (10%–15%)	Pulsed 775 nm (10%)
Sequence 3	Alexa Fluor 594	594 nm (5%–12%)	Pulsed 775 nm (15%)
Sequence 4	Alexa Fluor 555	555 nm (10%)	CW 660 nm (10%–15%)
Sequence 5	Alexa Fluor 488	488 nm (5%–10%)	CW 592 nm (10%–15%)

*Note: In this setup, 20% of the STED laser power in each channel is redirected to the Z-donut to enable 3D STED imaging. If only 2D STED is desirable, the STED depletion power may be further reduced to minimize photobleaching.*

**Critical:** All confocal sequences must be acquired before any STED imaging to prevent photobleaching from the high-power STED beams. For simultaneous acquisition of image stacks using multiple STED beams, use the “between stacks acquisition” settings. Do not acquire images “between lines” or “between frames”, as the CW 592 nm and CW 660 nm beams will photobleach red-shifted fluorophores.

**Critical:** For imaging of synaptic nanostructures in brain tissue with its three-dimensional complexity, 3D STED acquisition is necessary. Redirecting 20% of the laser power is sufficient to separate pre- and postsynaptic markers in the Z-orientation while acquiring individual Z-stacks at 150–180 nm intervals [28].

3. Using a 100× (1.4 NA) objective and a 1,024 × 1,024-pixel format, adjust the digital zoom (~4.5) to achieve a pixel size of ~20 nm. Always set the pixel size based on the channel with the highest expected resolution. When using the pulsed 775 nm laser, a 20–25 nm pixel size is sufficient to achieve the XY resolution in the range of 50 nm. A pixel size of 40 nm is appropriate for imaging with only the CW 592 nm and 660 nm STED beams.

**Critical:** Calibrate the resolution of the STED system using sub-diffraction beads of known separation, such as single- and dual-labeled GATTA nanorulers with 50, 70, or 140 nm spacing in XY (Table 2). Similarly, Z-resolution for each STED laser setting should be defined using 3D GATTA bead spacers, and STED power should be adjusted accordingly [6, 28].

**Table 2.** Full width at half maximum (FWHM) and physical distances measured using sub-diffraction beads

Fluorophore	Beads (23–40 nm diameter)	FWHM (single bead)	Physical two-bead separation	Validation
Atto 647N	GATTA STED 50R (gattaquant.com)	53 ± 3 nm	54 ± 1 nm (XY)	[6,28]
Atto 647N	GATTA 3D STED 90R (gattaquant.com)	Not defined	90 ± 2 nm (3D)	[28]
Atto 594	GATTA STED 140ROR (gattaquant.com)	52 ± 1 nm	66 ± 2 nm (XY)	[6,28]
Alexa Fluor 555	N/A	Not defined	Not defined	
Alexa Fluor 488	40 nm (505/515) beads, Thermo, catalog number: F8795	~80 nm	Not defined	[34]

4. For fluorescence lifetime imaging (FLIM)-based tau-STED, set HyD-X detectors to photon-counting to enable capturing single photons and identifying their lifetime profiles. Elimination of photons with short lifetimes will further improve image resolution. Adjust line/frame accumulations to ensure acquisition of at least 5–10 photons per pixel.

5. Following image acquisition, remove background (uncorrelated) photons from raw STED images by adjusting the “tau strength” parameter in the FAst Lifetime CONtrast (FALCON) module integrated into the Leica Stellaris 8 LAS X software. For images with an average photon count of 10 photons/pixel, tau strengths between 100 and 150 are appropriate. Once the tau strength is determined for each STED channel, it should be kept constant across all experiments.

**Critical:** Tau strength must be carefully optimized to reduce blur around cluster edges without distorting true structures or creating artifacts. While tau strength is closely linked to the distribution of photon lifetimes, which can be visualized using phasor plots, there is currently no expert-based approach in the Leica LAS X software that allows this to be done using image phasor plots alone. If the tau module is not available, it is recommended to engage the timed detectors to turn on 1–3 ns after the laser pulse.

6. The 16-bit images may be further processed in Fiji (ImageJ) by subtracting background, defined as the mean pixel intensities of the entire 1,024 × 1,024 frame, from every channel. Furthermore, adjust brightness to the maximum gray value within each Z-stack.

7. Convert processed 16-bit images to 8-bit images and apply a Gaussian blur of 0.5–0.8 pixels to each STED channel.

8. Processed images are then ready for further analyses, including quantifying cluster sizes, numbers, and intensities. For Z-stacks acquired with 3D tau-STED, it is advisable to reconstruct images in 3D to verify cluster size and synaptic localization. Commercially available software such as Imaris or Neurolucida 360 can be used for 3D rendering [28,34,36].

## Validation of protocol

This protocol was validated in both cultured cortical neurons and fixed brain cryosections to ensure robust and reproducible labeling of synaptic proteins, as described in detail in Akter et al. [28]. We performed a series of validation experiments to verify cryosection integrity, labeling reagent fidelity, and the effective resolution achieved with our STED configuration (Figure 4). By measuring the average pixel intensities of thresholded clusters in confocal mode, we observed consistent labeling of PSD-95 and Bassoon throughout the full thickness of the cryosections, indicating preserved tissue integrity and reliable imaging across tissue depths (Figure 4A–D). These experiments also revealed a ~10% reduction in puncta intensity from the top to the bottom of the sections. Nonetheless, synaptic clusters remained clearly distinguishable from background throughout the section thickness, with minimal or no artifacts at section edges.

We further evaluated the performance of conventional antibodies and nanobodies for in situ labeling by assessing the relative localization of active zone and PSD proteins, Bassoon, and PSD-95. Consistent with prior reports, antibody-based labeling of PSD-95 in 4% PFA-fixed tissue was suboptimal, resulting in poor colocalization with Bassoon clusters and an apparent absence of postsynaptic partners at many synapses [27,32]. However, this limitation was overcome using small, directly conjugated nanobodies, which reliably detected PSD-95 in apposition to Bassoon in PFA-fixed sections and enabled the visualization of most trans-synaptic pairs (Figure 4E–H). Glyoxal fixation improved antibody labeling for several synaptic targets; however, all nanobodies tested produced consistent pre- and postsynaptic labeling, independent of fixation method [28].

Finally, using the STED configuration described in Table 1, we reproducibly achieved ~50 nm lateral and ~100 nm axial resolution, validated using sub-diffraction fluorescent beads (Figure 4I–L, Table 2) [28]. Under these conditions, PSD-95 and Bassoon in cryosections exhibited full width at half maximum (FWHM) values of ~100–130 nm (Figure 4Mndash;O). Overall, employing all aspects of this protocol enabled reliable, multiplexed labeling and nanoscale visualization of synaptic nano-architecture at diverse synapses (VGluT1 vs. VGluT2) in brain cryosections using 3D STED imaging (Figure 4P).

This protocol, including its quantitative analyses, has been used and validated in the following research article:

• Akter et al. [28]. Combining nanobody labeling with STED microscopy reveals input-specific and layer-specific organization of neocortical synapses. PLoS Biol. 2025 Apr 4;23(4): e3002649. doi: 10.1371/journal.pbio.3002649. PMID: 40184426; PMCID: PMC12002638.

## General notes and troubleshooting

**General notes**

The cryosection preparation and immunolabeling steps described in this protocol are broadly applicable to multiple species, including mice, rats, and other animal models. With minor adjustments, the procedure can be adapted for immunolabeling synaptic proteins in primary cortical or hippocampal neuron cultures. Although this protocol was developed specifically to image the nano-organization of pre- and postsynaptic proteins in situ using STED, it is compatible with standard confocal microscopy. Importantly, the tissue preparation and immunolabeling steps are also compatible with expansion microscopy-based super-resolution approaches, which do not require specialized imaging equipment, as demonstrated in Akter et al. [28]. Although not specifically tested here, these steps should be readily adaptable to stochastic optical reconstruction microscopy (STORM) or other single-molecule localization techniques. Several key considerations are essential:

1. Tissue thickness and imaging quality: Thin cryosections allow more uniform penetration of labeling reagents and improve signal quality throughout the entire tissue thickness, enhancing quantitative image analysis. For STED nanoscopy, collecting sections directly onto coverslips reduces the distance between the tissue and the high-NA (1.4), low-working-distance (0.13 mm) objectives, while providing an even imaging surface, thereby reducing optical aberration and improving super-resolution imaging quality. A limitation of mounting sections directly onto coverslips is that labeling reagents penetrate primarily from the exposed surface, resulting in ~10% lower signal intensity at the bottom compared to the top of the section. However, this modest reduction does not compromise imaging, as synaptic clusters remain clearly distinguishable from background noise (Figure 4A–D).

2. Fixative choice: The fixative must be carefully matched to the labeling reagent. Glyoxal is preferred for many PSD proteins when using antibodies, while both 4% PFA or glyoxal are compatible with nanobodies (Figure 4E) [27,28].

3. Limitations of thin sections: Thin cryosections restrict imaging to short dendritic fragments and prevent identification of the parent cell body. Although slightly thicker sections can partially mitigate this limitation, increasing thickness reduces antibody penetration and negatively impacts XY resolution [24]. In our hands, 6 μm sections provide a practical compromise, exhibiting stable labeling intensity across the full section thickness while minimizing depth-dependent loss of XY resolution. Nonetheless, nanoclusters in these sections still suffer from anisotropy. This limitation may be addressed by sub-micron serial sectioning, similar to array tomography, which avoids thickness-related reduction in XY resolution and is more likely to resolve isotropic nanoclusters [37]. Investigators must weigh these trade-offs based on their experimental goals.

**Antibodies and nanobodies:** The choice of labeling reagents is critical for achieving high-quality synaptic labeling, particularly when targeting proteins in presynaptic terminals or postsynaptic densities, where densely packed proteins can mask epitopes. Nanobodies are approximately ten times smaller than antibodies (~3–4 nm) and therefore penetrate tissue more effectively across these crowded spaces. The combination of their small size and direct fluorophore conjugation makes nanobodies particularly well suited for super-resolution imaging of synapses. However, the major limitation of nanobodies is their currently limited repertoire, which restricts their use to a small subset of synaptic targets. As a result, combining nanobodies with conventional antibodies is often necessary to comprehensively examine the molecular nano-organization of diverse synapse types. Below are key considerations for selecting and applying these reagents effectively:

1. Presynaptic proteins: Antibodies against Bassoon, VGluT1, VGluT2, and SYT1 provide robust labeling of presynaptic terminals and active zones in both glyoxal- and 4% PFA–fixed tissues. Nanobodies against VGlutT1 and SYT1 produce labeling comparable to antibodies in 6 μm cryosections [28].

2. Postsynaptic density proteins: For PSD proteins such as PSD-95, the PSD-95 nanobody outperforms the PSD-95 antibody in PFA-fixed sections due to better epitope accessibility (Figure 4E–G) [28,38]. Therefore, if simultaneous labeling of active zones and PSDs is a goal, nanobodies should be reserved for proteins in the PSD due to the high protein crowding. If antibodies must be used, glyoxal fixation is strongly recommended [27,28]. Nanobodies remain preferable in all cases because of their small size and direct fluorophore conjugation that reduces linkage error, further enhancing resolution [39]. Furthermore, nanobodies are species independent, enabling highly multiplexed imaging (Figure 4P) [26].

**Multiplexed labeling:** Although this protocol describes imaging up to five channels simultaneously (Figure 4P), it can be expanded to additional synaptic proteins depending on the imaging system and reagent availability. Because nanobodies are species-independent, combining them with conventional antibodies supports highly multiplexed labeling [26,28]. However, increasing the number of fluorophores raises the risk of spectral overlap and channel crosstalk. Five channels likely represent the upper limit for most conventional systems. For experiments requiring more channels, investigators should use imaging systems or computational algorithms capable of spectral unmixing to reliably separate overlapping emission spectra [40,41]. Although the Leica Stellaris 8 system supports spectral unmixing using photon lifetime measurements, this should be performed with caution using proper reference spectra to avoid causing artifacts.

**Troubleshooting**

**A. Perfusion**

**Problem 1:** Improper perfusion of brain tissue. This problem is evident when the mouse fails to tense and become rigid within 30 s of fixative perfusion, and the brain remains reddish-pink instead of turning opaque white.

Possible causes: There are several underlying causes, including incorrect placement of the needle into the heart muscle (e.g., missing the left ventricle, entering too deeply, or puncturing through to the opposite side), the presence of air bubbles in the tubing that prevent flow of ACSF and fixative, or improperly prepared perfusion solutions.

Solutions: Ensure that all air is completely flushed from both the ACSF and fixative lines before starting the perfusion. Proper needle placement is critical: insert the needle through the apex of the left ventricle and keep it angled upward toward the left atrium, taking care not to advance it too deeply—stop just beyond the hemostats to avoid puncturing the opposite side of the heart. Finally, confirm that all solutions are prepared exactly according to the recipes and are made fresh just before the experiment. If the solutions are coming out of the nostrils, this is an indication of either a fast perfusion rate or the incorrect placement of the needle.

**B. Cryosectioning**

**Problem 1:** Sections roll up, not cutting cleanly as flat sheets.

Possible cause: The anti-roll plate is improperly positioned, causing the sections to curl as they come off the blade.

Solution: Carefully adjust the anti-roll plate so that its bottom edge aligns precisely with the cutting edge of the blade. Make small, incremental adjustments until the sections consistently come off the knife as smooth, flat sheets.

**Problem 2:** Cryosections stick to the anti-roll plate.

Possible causes: Static buildup on the anti-roll plate or the chamber has become too warm.

Solutions: Use a Zerostat anti-static instrument to remove the static from the anti-roll plate. To bring the chamber temperature down, close the door to the cryostat chamber and allow the chamber to cool for 20–30 min.

**Problem 3:** Sections are tearing.

Possible causes: Debris from OCT or tissue is sticking to the blade or anti-roll plate.

Solution: Clean both the blade and anti-roll plate by using a large paint brush.

**C. Immunolabeling/mounting**

**Problem 1:** Weak or no staining of synaptic proteins.

Possible causes: This may result from using the wrong fixative during perfusion, using antibody/nanobody concentrations that are too low, or leakage of primary or secondary antibodies due to an incomplete seal between the CoverWell and the coverslip.

Solutions: Ensure that brain tissue is perfused with glyoxal fixative (Recipe 4) when labeling PSD proteins with antibodies such as PSD-95 [27,28]. If PFA fixation is required, consider using nanobodies, although their availability for specific targets is still limited. Antigen retrieval can be used to improve antigen accessibility, and tissue-clearing approaches such as CUBIC or iDISCO can enhance fluorescence intensity and antibody penetration by removing lipids [22,23]. However, this may be less desirable for super-resolution imaging because mismatches between the refractive indices of the tissue solvents, mounting medium, and high–NA objective immersion oil can introduce spherical aberration and limit resolution [42]. Increasing antibody concentration may also improve the signal, though it can raise background levels. Finally, verify that the CoverWell forms a tight seal with the coverslip to prevent leakage of labeling reagents.

**Problem 2:** High background and a low S/N ratio, which reduces resolution and can introduce artifacts when using the STED imaging approach.

Possible causes: Insufficient blocking or high concentration of primary or secondary antibodies.

Solutions: Extend the blocking step up to 48 h to suppress nonspecific binding. Alternatively, optimize the blocking buffer (Recipe 7) by adding 1%–5% BSA and ensuring that the serum used in the blocking solution matches the host species of the secondary antibody. Reduce the concentration of primary or secondary antibodies or shorten the immunolabeling incubation duration to minimize background while maintaining adequate signal.

**Problem 3:** Spotty labeling with areas of both high and low signal

Potential cause: Formation of bubbles or microbubbles in the primary or secondary antibody solutions, leading to uneven contact between the reagents and the tissue.

Solution: Ensure that sufficient antibody solution (300 μL) is added to each CoverWell and that a tight seal forms between the CoverWell and the coverslip. When applying the coverslip, lower it gently rather than dropping it onto the antibody solution, as sudden placement can introduce foaming and microbubbles that disrupt uniform labeling.

**D. STED imaging and tissue processing**

**Problem 1:** No apparent STED/super-resolution effect

Potential causes: Misaligned excitation and STED lasers, insufficient STED laser power, or low fluorescence signal leading to poor S/N ratio.

Solutions: Verify laser alignment using 80 nm gold beads, ensuring that the excitation beam passes directly through the center of the STED depletion-beam donut. Increase the STED laser power as needed to achieve sufficient depletion. Confirm that the sample produces a strong, clean fluorescence signal in conventional confocal mode. If the confocal image shows high background or low signal, optimize the staining protocol first to improve signal quality before attempting STED imaging.

**Problem 2:** Photobleaching.

Potential causes: Scanning speed is too low, STED laser power is too high, or incompatible dyes.

Solutions: Photobleaching is often caused by high-intensity STED lasers, especially when using continuous-wave (CW) depletion beams. Therefore, when imaging multiple STED channels, acquire channels that use the pulsed 775 nm STED beam first, followed by those using the CW 660 nm beam, and finally those using the CW 592 nm beam (Table 1). To reduce bleaching, increase the scanning speed to shorten pixel dwell time. When possible, use the resonant scanner (8,000 Hz), especially for bright samples with high S/N ratios [34]. If a resonant scanner is not available, increase the speed of conventional scanning and reduce the number of line accumulations, as repeated line scans can significantly increase bleaching at lower scan speeds. Alternatively, lower the STED laser power. This is particularly effective on systems with FLIM-enabled tau-STED (e.g., Leica Stellaris), where resolution can be further improved by removing uncorrelated photons [28]. Finally, ensure that only STED-compatible dyes are used: those with high photostability that can tolerate intense depletion beams (see Table 1 and Table S1) [6,28,34,35].

## Supplementary information

The following supporting information can be downloaded here:

1. Table S1. Validated antibodies and nanobodies for immunolabeling synaptic nanoarchitecture for STED microscopy

## References

[1] Biederer T., Kaeser P. S. and Blanpied T. A. (2017). Transcellular Nanoalignment of Synaptic Function. Neuron. 96(3): 680 696 696. 10.1016/j.neuron .2017.10.006 29096080 PMC5777221

[2] Fukata Y., Fukata M., MacGillavry H. D., Nair D. and Hosy E. (2024). Celebrating the Birthday of AMPA Receptor Nanodomains: Illuminating the Nanoscale Organization of Excitatory Synapses with 10 Nanocandles. J Neurosci. 44(23): e2104232024. 10.1523/JNEUROSCI.2104-23 .2024 PMC11154862 38839340

[3] MacGillavry H. D., Song Y., Raghavachari S. and Blanpied T. A. (2013). Nanoscale scaffolding domains within the postsynaptic density concentrate synaptic AMPA receptors. Neuron. 78(4): 615 –-622. 10.1016/j.neuron .2013.03.009 23719161 PMC3668352

[4] Crosby K. C., Gookin S. E., Garcia J. D., Hahm K. M., Dell’Acqua M. L. and Smith K. R. (2019). Nanoscale Subsynaptic Domains Underlie the Organization of the Inhibitory Synapse. Cell Rep. 26(12): 3284 3297 3297 e3283. 10.1016/j.celrep .2019.02.070 30893601 PMC6529211

[5] Goncalves J., Bartol T. M., Camus C., Levet F., Menegolla A. P., Sejnowski T. J., Sibarita J. B., Vivaudou M., Choquet D., Hosy E., .(2020). Nanoscale co-organization and coactivation of AMPAR, NMDAR, and mGluR at excitatory synapses. Proc Natl Acad Sci USA. 117(25): 14503 14511 14511. 10.1073/pnas.1922563117 32513712 PMC7321977

[6] Hruska M., Cain R. E. and Dalva M. B. (2022). Nanoscale rules governing the organization of glutamate receptors in spine synapses are subunit specific. Nat Commun. 13(1): 920 10.1038/s41467-022-28504-4 35177616 PMC8854560

[7] Kellermayer B., Ferreira J. S., Dupuis J., Levet F., Grillo-Bosch D., Bard L., Linares-Loyez J., Bouchet D., Choquet D., Rusakov D. A., .(2018). Differential Nanoscale Topography and Functional Role of GluN2-NMDA Receptor Subtypes at Glutamatergic Synapses. Neuron 100(1): 106 119 119 e107. 10.1016/j.neuron .2018.09.012 30269991

[8] Nair D., Hosy E., Petersen J. D., Constals A., Giannone G., Choquet D. and Sibarita J. B. (2013). Super-resolution imaging reveals that AMPA receptors inside synapses are dynamically organized in nanodomains regulated by PSD95. J Neurosci. 33(32): 13204 13224 13224. 10.1523/JNEUROSCI.2381-12 .2013 23926273 PMC6619720

[9] Tang A. H., Chen H., Li T. P., Metzbower S. R., MacGillavry H. D. and Blanpied T. A. (2016). A trans-synaptic nanocolumn aligns neurotransmitter release to receptors. Nature. 536(7615): 210 214 214. 10.1038/nature19058 27462810 PMC5002394

[10] Ramsey A. M., Tang A. H., LeGates T. A., Gou X. Z., Carbone B. E., Thompson S. M., Biederer T. and Blanpied T. A. (2021). Subsynaptic positioning of AMPARs by LRRTM2 controls synaptic strength. Sci Adv. 7(34): eabf3126. https://doi.org/10.1126/sciadv.abf3126 PMC8378824 34417170

[11] Olah S. S., Kareemo D. J., Buchta W. C., Sinnen B. L., Miller C. N., Actor-Engel H. S., Gookin S. E., Winborn C. S., Kleinjan M. S., Crosby K. C., .(2023). Acute reorganization of postsynaptic GABA(A) receptors reveals the functional impact of molecular nanoarchitecture at inhibitory synapses. Cell Rep. 42(11): 113331 10.1016/j.celrep .2023.113331 37910506 PMC10782565

[12] Grant S. G. (2007). Toward a molecular catalogue of synapses. Brain Res Rev. 55(2): 445 449 449. 10.1016/j.brainresrev .2007.05.003 17572504

[13] O’Rourke N. A., Weiler N. C., Micheva K. D. and Smith S. J. (2012). Deep molecular diversity of mammalian synapses: why it matters and how to measure it. Nat Rev Neurosci. 13(6): 365 379 379. 10.1038/nrn3170 22573027 PMC3670986

[14] van Oostrum M., Blok T. M., Giandomenico S. L., Tom Dieck S., Tushev G., Furst N., Langer J. D. and Schuman E. M. (2023). The proteomic landscape of synaptic diversity across brain regions and cell types. Cell. 186(24): 5411 5427 5427 e5423. 10.1016/j.cell .2023.09.028 37918396 PMC10686415

[15] Peng H., Xie P., Liu L., Kuang X., Wang Y., Qu L., Gong H., Jiang S., Li A., Ruan Z., .(2021). Morphological diversity of single neurons in molecularly defined cell types. Nature. 598(7879): 174 181 181. 10.1038/s41586-021-03941-1 34616072 PMC8494643

[16] Siletti K., Hodge R., Mossi Albiach A., Lee K. W., Ding S. L., Hu L., Lonnerberg P., Bakken T., Casper T., Clark M., .(2023). Transcriptomic diversity of cell types across the adult human brain. Science. 382(6667): eadd7046. https://doi.org/10.1126/science.add7046 37824663

[17] Getz A. M., Ducros M., Breillat C., A. Lampin-Saint-Amaux, Daburon S., Francois U., Nowacka A., Fernandez-Monreal M., Hosy E., Lanore F., .(2022). High-resolution imaging and manipulation of endogenous AMPA receptor surface mobility during synaptic plasticity and learning. Sci Adv. 8(30): eabm5298. https://doi.org/10.1126/sciadv.abm5298 PMC9328687 35895810

[18] Velicky P., Miguel E., Michalska J. M., Lyudchik J., Wei D., Lin Z., Watson J. F., Troidl J., Beyer J., Ben-Simon Y., .(2023). Dense 4D nanoscale reconstruction of living brain tissue. Nat Methods. 20(8): 1256 1265 1265. 10.1038/s41592-023-01936-6 37429995 PMC10406607

[19] Andrews N. P., Boeckman J. X., Manning C. F., Nguyen J. T., Bechtold H., Dumitras C., Gong B., Nguyen K., van der List D., Murray K. D., .(2019). A toolbox of IgG subclass-switched recombinant monoclonal antibodies for enhanced multiplex immunolabeling of brain. eLife 8: e43322. https://doi.org/10.7554/eLife.43322 PMC6377228 30667360

[20] Dong J. X., Lee Y., Kirmiz M., Palacio S., Dumitras C., Moreno C. M., Sando R., Santana L. F., Sudhof T. C., Gong B., .(2019). A toolbox of nanobodies developed and validated for use as intrabodies and nanoscale immunolabels in mammalian brain neurons. eLife 8: e48750. https://doi.org/10.7554/eLife.48750 PMC6785268 31566565

[21] Micheva K. D., Busse B., Weiler N. C., N. O’Rourke and Smith S. J. (2010). Single-synapse analysis of a diverse synapse population: proteomic imaging methods and markers. Neuron. 68(4): 639 653 653. 10.1016/j.neuron .2010.09.024 21092855 PMC2995697

[22] Renier N., Wu Z., Simon D. J., Yang J., Ariel P. and Tessier-Lavigne M. (2014). iDISCO: a simple, rapid method to immunolabel large tissue samples for volume imaging. Cell. 159(4): 896 910 910. 10.1016/j.cell .2014.10.010 25417164

[23] Susaki E. A., Tainaka K., Perrin D., Kishino F., Tawara T., Watanabe T. M., Yokoyama C., Onoe H., Eguchi M., Yamaguchi S., .(2014). Whole-brain imaging with single-cell resolution using chemical cocktails and computational analysis. Cell. 157(3): 726 739 739. 10.1016/j.cell .2014.03.042 24746791

[24] Schermelleh L., Ferrand A., Huser T., Eggeling C., Sauer M., Biehlmaier O. and Drummen G. P. C. (2019). Super-resolution microscopy demystified. Nat Cell Biol. 21(1): 72 84 84. 10.1038/s41556-018-0251-8 30602772

[25] Sheng M. and Hoogenraad C. C. (2007). The postsynaptic architecture of excitatory synapses: a more quantitative view. Annu Rev Biochem. 76: 823 847 847. 10.1146/annurev.biochem .76.060805.160029 17243894

[26] Unterauer E. M., Shetab Boushehri S., Jevdokimenko K., Masullo L. A., Ganji M., Sograte-Idrissi S., Kowalewski R., Strauss S., Reinhardt S. C. M., Perovic A., .(2024). Spatial proteomics in neurons at single-protein resolution. Cell. 187(7): 1785 1800 1800 e1716. 10.1016/j.cell .2024.02.045 38552614

[27] Konno K., Yamasaki M., Miyazaki T. and Watanabe M. (2023). Glyoxal fixation: An approach to solve immunohistochemical problem in neuroscience research. Sci Adv. 9(28): eadf7084. https://doi.org/10.1126/sciadv.adf7084 PMC10348680 37450597

[28] Akter Y., Jones G., Daskivich G. J., Shifflett V., Vargas K. J. and Hruska M. (2025). Combining nanobody labeling with STED microscopy reveals input-specific and layer-specific organization of neocortical synapses. PLoS Biol. 23(4): e3002649. 10.1371/journal.pbio .3002649 PMC12002638 40184426

[29] Feng G., Mellor R. H., Bernstein M., Keller-Peck C., Nguyen Q. T., Wallace M., Nerbonne J. M., Lichtman J. W. and Sanes J. R. (2000). Imaging neuronal subsets in transgenic mice expressing multiple spectral variants of GFP. Neuron. 28(1): 41 51 51. 10.1016/s0896-6273 (00)00084-2 11086982

[30] Schindelin J., Arganda-Carreras I., Frise E., Kaynig V., Longair M., Pietzsch T., Preibisch S., Rueden C., Saalfeld S., Schmid B., .(2012). Fiji: an open-source platform for biological-image analysis. Nat Methods. 9(7): 676 682 682. 10.1038/nmeth.2019 22743772 PMC3855844

[31] Wu J., Cai Y., Wu X., Ying Y., Tai Y. and He M. (2021). Transcardiac Perfusion of the Mouse for Brain Tissue Dissection and Fixation. Bio Protoc. 11(5): e3988. 10.21769/BioProtoc.3988 PMC8005872 33796622

[32] Fang T., Lu X., Berger D., Gmeiner C., Cho J., Schalek R., Ploegh H. and Lichtman J. (2018). Nanobody immunostaining for correlated light and electron microscopy with preservation of ultrastructure. Nat Methods. 15(12): 1029 1032 1032. 10.1038/s41592-018-0177-x 30397326 PMC6405223

[33] Huang W. M., Gibson S. J., Facer P., Gu J. and Polak J. M. (1983). Improved section adhesion for immunocytochemistry using high molecular weight polymers of L-lysine as a slide coating. Histochemistry. 77(2): 275 279 279. 10.1007/BF00506570 6188729

[34] Hruska M., Henderson N., Le Marchand S. J., Jafri H. and Dalva M. B. (2018). Synaptic nanomodules underlie the organization and plasticity of spine synapses. Nat Neurosci. 21(5): 671 682 682. 10.1038/s41593-018-0138-9 29686261 PMC5920789

[35] Jeong S., Widengren J. and Lee J. C. (2021). Fluorescent Probes for STED Optical Nanoscopy. Nanomaterials(Basel). 12(1): 21 10.3390/nano12010021 35009972 PMC8746377

[36] Hruska M. and Dalva M. B. (2012). Ephrin regulation of synapse formation, function and plasticity. Mol Cell Neurosci. 50(1): 35 44 44. 10.1016/j.mcn .2012.03.004 22449939 PMC3631567

[37] Micheva K. D., Gong B., Collman F., Weinberg R. J., Smith S. J., Trimmer J. S. and Murray K. D. (2023). Developing a Toolbox of Antibodies Validated for Array Tomography-Based Imaging of Brain Synapses. eNeuro. 10(12): ENEURO.0290-0223.2023. 10.1523/ENEURO.0290-23 .2023 PMC10748464 37945352

[38] Kilisch M., Gere-Becker M., Wustefeld L., Bonnas C., Crauel A., Mechmershausen M., Martens H., Gotzke H., Opazo F. and Frey S. (2023). Simple and Highly Efficient Detection of PSD95 Using a Nanobody and Its Recombinant Heavy-Chain Antibody Derivatives. Int J Mol Sci. 24(8): 7294 10.3390/ijms24087294 37108454 PMC10138605

[39] K. Queiroz Zetune Villa Real, Mougios N., Rehm R., Sograte-Idrissi S., Albert L., Rahimi A. M., Maidorn M., Hentze J., Martinez-Carranza M., Hosseini H., .(2023). A Versatile Synaptotagmin-1 Nanobody Provides Perturbation-Free Live Synaptic Imaging And Low Linkage-Error in Super-Resolution Microscopy. Small Methods. 7(10): e2300218. https://doi.org/10.1002/smtd.202300218 37421204

[40] Lippincott-Schwartz J. (2018). Uncovering Theorganelle Interactome: Dynamic Imaging of Multiple Organelles. Biophys J. 114(3): 367 a. 10.1016/j.bpj .2017.11.2034

[41] Valm A. M., Cohen S., Legant W. R., Melunis J., Hershberg U., Wait E., Cohen A. R., Davidson M. W., Betzig E. and Lippincott-Schwartz J. (2017). Applying systems-level spectral imaging and analysis to reveal the organelle interactome. Nature. 546(7656): 162 167 167. 10.1038/nature22369 28538724 PMC5536967

[42] Richardson D. S. and Lichtman J. W. (2015). Clarifying Tissue Clearing. Cell. 162(2): 246 257 257. 10.1016/j.cell .2015.06.067 26186186 PMC4537058

